# Vitamins (A, C and E) and oxidative status of hemodialysis patients treated with HFR and HFR-Supra

**DOI:** 10.1186/s12882-016-0315-6

**Published:** 2016-08-26

**Authors:** Simonetta Palleschi, Paolo M. Ghezzi, Giuseppe Palladino, Barbara Rossi, Marino Ganadu, Domenica Casu, Maria Cossu, Giovanni Mattana, Antonio Maria Pinna, Bruno Contu, Tonina Ghisu, Alessandro Monni, Luana Gazzanelli, Maria Cristina Mereu, Franco Logias, Mario Passaghe, Alessandro Amore, Piergiorgio Bolasco, on behalf of the Sardinian Study Group, Irene Sitzia, Marialuisa Caiazzo, Luciangela Calvisi, Angelo Piras, Annalisa Sini, Emiliana Sulis, Maria Rosaria Scalas, Pierluigi Spiga, Andrea Martorana, Sabina Fancello, Elisa Loiacono, Luisa Sereni

**Affiliations:** 1 Department of Hematology, Oncology and Molecular Medicine, Istituto Superiore di Sanità, Rome, Italy; 2 Medical Scientific Consultant of Bellco s.r.l. Company, Mirandola, Italy; 3 Bellco s.r.l Company, Mirandola, Italy; 4 Ospedale A.Segni, Ozieri, Italy; 5 Ospedale Civile, Alghero, Italy; 6 Ospedale SS. Annunziata, Sassari, Italy; 7 Ospedale S. Francesco, Nuoro, Italy; 8 Ospedale S. Martino, Oristano, Italy; 9 Ospedale N.S. della Mercede, Lanusei, Italy; 10 ASL 3, Macomer, Italy; 11 Ospedale SS. Trinità, Cagliari, Italy; 12 Ospedale P. Merlo, La Maddalena, Italy; 13 Ospedale N.S. di Bonaria, S. Gavino Monreale, Italy; 14 Ospedale P. Dettori, Tempio Pausania, Italy; 15 Ospedale Infantile Regina Margherita, Torino, Italy; 16 Territorial Unit of Nephrology and Dialysis , ASL 8 of Cagliari, Cagliari, Italy

**Keywords:** Oxidative stress, Hemodiafiltration, Antioxidants, Vitamins, Adsorption, HFR, High permeability

## Abstract

**Background:**

Hemodiafiltration with on-line endogenous reinfusion (HFR) is an extracorporeal dialytic method that combines diffusion, convection and adsorption. HFR-Supra (HFR-S) is a second-generation system with increased convective permeability and adsorption capability. Previous studies suggested that HFR reduces oxidative stress compared to standard haemodialysis. The principal aim of the present study was to compare antioxidant vitamins behavior and oxidative status of hemodialysis patients treated with HFR and HFR-S.

**Methods:**

The study was designed as a multicenter, randomized, crossover trial. Forty-one patients were recruited from 19 dialysis centers and after a 4-month washout stabilization period in on-line hemodiafiltration (ol-HDF), each patient was randomized to a sequence of treatments (HFR-S followed by HFR or viceversa) with each treatment applied over 6 months. Plasma levels of Advanced Oxidation Protein Products, Total Antioxidant Status, vitamins C, A and E and their ligands (Retinol Binding Protein and total lipids) were measured at baseline and at the end of each treatment period.

**Results:**

Results show that the higher convective permeability of HFR-S with respect to HFR did not produce additional beneficial effects on the patients’ oxidative status, a slight decrease of both Vitamin A and Retinol Binding Protein being the only difference registered in the long-term. However, as compared to ol-HDF, both the re-infusive techniques allowed to reduce the intradialytic loss of Vitamin C and, in the long-term, improve the patients’ oxidative status and increase Retinol Binding Protein plasma values. No significant differences were found between the Vitamin C concentration of pre- and post cartridge UF neither in HFR-S nor in HFR showing that the sorbent resin does not adsorb Vitamin C.

**Conclusion:**

HFR-S and HFR are almost equivalent in term of impact on antioxidant vitamins and oxidative status of hemodialysis patients. Nonetheless, as compared to ol-HDF, both treatments produced a sensible sparing of Vitamin C and may represent a new approach for reducing oxidative stress and related complications in dialysis patients. Long-term effects of re-infusive treatments on patients’ cardiovascular morbidity and mortality need to be evaluated.

**Trial registration:**

ClinicalTrials.gov Identifier NCT01492491, retrospectively registered in 10 December 2011.

## Background

Oxidative stress and inflammation are common occurrences in end stage renal disease (ESRD) and are believed to play an important role in the excess cardiovascular morbidity and mortality of these patients [1]. Oxidative stress in ESRD is due to both enhanced oxidative capacity and reduced antioxidant defenses, the latter including impaired enzyme activities and decreased levels of antioxidant vitamins [1–3]. Vitamin deficiencies in ESRD may originate from diet restriction, reduced absorption by medications and co-morbidities, uremia-related alterations of metabolic pathways and intradialytic losses [3]. Vitamin C (Vit C) is the most abundant and effective water-soluble antioxidant in human plasma. Vit. C deficiency is very common in dialysis patients mainly because of low dietary intake, losses during dialysis and accelerated catabolism [4–6]. Low levels of Vit C may have clinical consequences and associations with increased cardiovascular morbidity and mortality have been found [7, 8]. Vitamin E (Vit E) and Vitamin A (Vit A) are two fat-soluble plasma antioxidants and are transported in plasma by lipoproteins and retinol binding protein (RBP), respectively. Although inconsistent levels of Vit E levels have been reported in uremic patients [3], the administration of this vitamin has been suggested as a promising therapeutic strategy to limit both oxidative stress and its clinical consequences in hemodialysis patients; however, results obtained to date are contradictory [3, 9, 10]. Vit. A levels are generally increased in ESRD patients, mainly due to homeostatic dysregulation of its plasma carrier, the retinol binding protein (RBP) [3, 11]. Nonetheless, in ESRD patients relatively higher Vit. A concentrations are associated with a survival advantage [12, 13]. The molecular mechanisms underlying such an association have not yet been elucidated, albeit a role in the inhibition of inflammatory response has been hypothesized [12].

The enhanced oxidative capacity in uremia is due, at least in part, to systemic microinflammation and up-regulation of superoxide-producing enzymes [1]. This chronic condition results in structural modification of many plasma components, mainly protein and lipids [14]. These modified molecules, even of large size, might themselves become pro-inflammatory so ultimately leading to a further increase of oxidative stress. The removal of large uremic solutes, by breaking this vicious cycle, can therefore contribute to decrease oxidative stress in ESRD. It is known that large-size uremic solutes can more efficiently be removed by convective dialysis modalities, such as haemodiafiltration (HDF), than by standard haemodialysis (HD). On-line HDF (ol-HDF), performed using high-flux biocompatible membrane and high-quality ultrapure dialysate, has been reported to provide superior reduction in inflammation and oxidative stress than high-flux HD [15, 16]. However, convective techniques may also cause negative effects, including the loss of water-soluble antioxidants and amino acids, which cannot be replaced by reinfusion of the dialysate. In particular, it has been calculated that convective transport in ol-HDF is responsible of one-third of Vit. C loss during dialysis, so limiting the advantages of this techniques in decreasing oxidative stress [6].

**Fig. 1:**
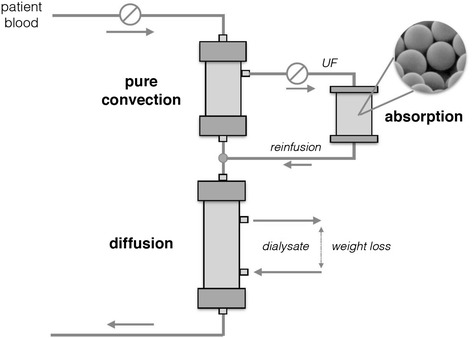
Schematic representation of HFR

Hemodiafiltration with endogenous reinfusion (HFR) is a kind of HDF that utilizes separated convection, diffusion and adsorption (Fig. 1) [17]. The system consist of a two-stage filter to separate convection from diffusion, combined with a sorbent cartridge to regenerate blood ultrafiltrate (UF). The regenerated ultrafiltrate, ie the plasmatic water deprived of those compounds which had bound to the sorbent resin, is reinfused back to the patient between the first and second stage of the filter. The sorbent cartridge contains styrene divinylbenzene (SDVB) hydrophobic resin which has a high affinity for different uremic toxins, cytokines and inflammatory mediators [18–21] while it does not retain nutrients like albumin and amino acids [22–24]. Hence, this technique allows the removal of medium/large uremic solutes by avoiding at the same time the loss of important nutrients. Previous studies showed that HFR has a better impact on both inflammation and oxidative stress than HD [25–28], with an efficacy similar to that of ol-HDF in the long-term reduction of inflammatory markers [29]. Notably, HFR architecture permits to increase convective membrane permeability up to a level which, due to the risk of severe nutrients losses and consequent malnutrition, is not achievable with standard HDF techniques. In the new HFR SUPRA (HFR-S) configuration, the permeability of the first convective chamber was incremented with a new polyethersulfone (PES) hyper high-flux membrane, Synclear 0.2 (Table 1). Uf proteomic analysis showed that Synclear 0.2, as compared to a PES high-flux membrane, allows a better extraction of middle-high molecular weight solutes and protein bound uremic toxins [30, 31]. Based on all the above, one can thus hypothesize that the increased depurative capacity of HFR-S might have a positive impact on the oxidative status of dialysis patients. The present study was designed to verify this hypothesis by comparing HFR and HFR-S treatments as for their short- and long-term effects on plasma levels of Advanced Protein Oxidation Products (AOPP), Total Antioxidant Status (TAS) and antioxidant vitamins C, A, E and their ligands (Retinol Binding Protein, RBP, and total lipids for Vit A and E, respectively). Moreover, since the UF re-infusion would allow sparing (micro)nutrients such as Vit C thereby potentially beneficial effects on patients (anti)oxidative status are produced, a secondary aim of the study was to compare the two re-infusive techniques with the ol-HDF, used in the run-in phase.

**Table 1:** Characteristics of HFR and HFR-S filters

	Filter section	HFR	HFR-S
Membrane Type (surface)	convective	PES HF (0.7 m^2^)	PES HHF (0.7 m^2^)
*Sterilization mode: gamma rays*	diffusive	PES LF (1.7 m^2^)	PES LF (1.7 m^2^)
Kuf (mL/h/mmHg)	convective	25	39
	diffusive	13	13
Albumin^a^ sieving coefficient	convective	0.002	0.2
	diffusive	<0.0001	<0.0001
Adsorbent cartridge (volume) Sterilization mode: dry steam		SDVB (40 mL)	SDVB (80 mL)

*PES* Polyethersulfone (*LF* Low flux, *HF* high flux, *HHF* Hyper high flux)

*SDVB* Styrene Divinylbenzene

^a^Bovin Serum Albumin

Finally, we evaluated the sieving coefficient (SC-like) of vit C through the convective membranes and its adsorption on the SDVB resin cartridges at both the start and the end of HFR-S and HFR single sessions.

## Methods

### Study design and participant characteristics

**Fig. 2:**
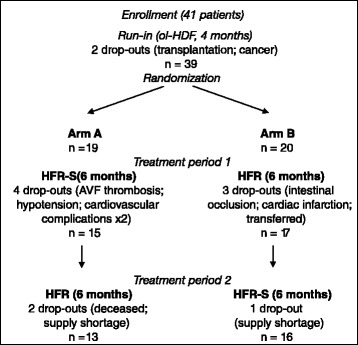
Study flow-chart

The study was designed as a prospective, multicenter, crossover trial with centralized randomization (Fig. 2). Between July and December 2011, forty-one ESRD patients were recruited from 19 territorial Dialysis Units of the National Public Health Care Service of Sardinia (Italy); their mean age was 67.0 ± 14.2 years (min 21, max 86); mean dialysis vintage was 101.8 ± 82.2 months. The total duration of the study was 16 months: after a 4-month run-in period in post-dilution ol-HDF by a hollow-fiber dialyzers (PHYLTHER, Bellco S.r.l., Mirandola, Italy) with 1.7 m^2^ PES HF membrane, each patient was randomized to a sequence of treatments (HFR-S followed by HFR or viceversa) with each treatment applied over 6 months. The randomization procedure was stratified by Center. Twenty-nine patients completed the study: two patients withdrew during the run-in period, further seven and three patients during the first and second treatment period, respectively (Fig. 2). Due to the earthquake that struck Mirandola district in 2012, some logistical problems arose in properly storing the plasma aliquots for AOPP an TAS measurements collected at the end of the second treatment period. Hence, only the values up to the first treatment period are available for these variables.

Inclusion criteria were dialysis vintage longer than 6 months, age > 18 years and well functioning vascular access without recirculation (Qb ≥ 300 mL/min). Exclusion criteria were: residual diuresis > 300 mL/day; significant acute or chronic inflammatory comorbidities; non-renal related anemia; alcohol or drugs abuse; malignant neoplasms; hemoglobinopathy or mielopathy; pregnancy. The causes of ESRD were diabetes (21 %), glomerulonephritis (16 %), interstitial nephrits (9 %), angionephrosclerosis (30 %), polycystic kidney disease (5 %), undeterminated etiology nature (19 %). The majority of patients followed a drug regimen for the control of blood pressure, anemia and secondary hyperparathyroidism. All the subjects were warned to avoid antioxidant supplements thorough all the study.

The extracorporeal dialysis treatment was performed three times per week. All 19 participating Centers made use of ultrapure, sterile and pyrogen-free dialysis solutions checked once monthly by cultural and endotoxins confirmation. Dialysate fluids were produced by a bi-osmosis and purified by daily nocturnal thermal disinfection of the piping (≥90 °C) [32–34].

### Preparation of collection tubes for Vit C analysis

To prevent the *ex vivo* degradation of Vit C, a reducing agent (dithioerythritol, DTE, 10 mmol/L final concentration in blood) was added in advance to the collection tubes. 10X DTE in physiological saline solution was prepared just before use, made sterile by filtration (0.22 μm pore size) and added to the tubes for blood and UF withdrawal (0.3 mL for each 3 mL heparinised gel-containing vacutainer and 0.2 mL for each 2 mL amber microcentrifuge tube, respectively). An insulin syringe was used to preserve the vacutainer void. The entire procedure was performed in a sterile environment and was highly reproducible (CV = 1.6 %). The tubes with the additive were stored at 4 °C until use (max 30 days). Preliminary experiments showed that the amount of DTE added did not affected blood cells integrity nor blood rheology up to at least 60 min from the collection, effectively preserving the Vit C content of either plasma or UF up to at least 90 days when specimens were stored at −25 °C [35].

### Blood and UF sampling and processing

Blood and pre- and post-cartridge UF samples were collected at any time of the study concomitantly with routine blood tests, both at the beginning and at the end of a first weekly dialysis session. Blood was collected in heparin gel-containing tubes (for vitamins’ assays) and EDTA tubes (for RBP, AOPP and TAS assays) and centrifuged within 30 min from the collection. Gel-containing centrifuged tubes, separated EDTA plasma and UF samples were then immediately frozen (−20 °C). Within 10 days from the collection, the samples were transferred in dry ice to the laboratory where they were stored at −80 °C until analysis (30 days max).

### AOPP and TAS assays

Plasma AOPP levels were measured by spectrophotometry on a microplate reader (Bio-Rad 680 XR, Hercules, CA, USA) calibrated with chloramine-T solutions (Sigma-Aldrich, Saint Louis, MO, USA) in the presence of potassium iodide [36]. Absorbance of the reaction mixture was read at 340 nm against a blank solution. Results were expressed as μmol/L of chloramine-T equivalents.

Plasma TAS levels were measured by a commercial colorimetric assay (TAS Randox, County Antrim, UK).

### Vit._s_ C, A, E assays

The determination of Vit_s_ C, A and E was carried out by reverse-phase high performance liquid chromatography (Varian model 9010, Varian Medical Systems, Palo Alto, CA, USA) with UV and FL detection, according to previously published methods with some modifications [37, 38]. For Vit C assay, plasma and UF aliquots were extracted with an equal volume of 10 % metaphosphoric acid and the acidic extracts were then separated on a Synergy Polar-RP column (150 mm × 4.6 mm, 4 μm particle size, Phenomenex, Torrance, CA, USA), using 50 mM potassium phosphate buffer at pH 2.5 as mobile phase. For vit._s_ A and E assay, plasma aliquots were supplemented with the internal standard (6 μmol/L retinyl acetate) and extracted with an equal volume of a mixture of ethyl acetate-butanol (1:1). The separation of organic extracts was then performed on a fully end-capped C18 (150 mm × 4.6 mm, 5 μm particle size, Spherisorb ODS2, Waters Corporation, Milford, MA, USA) using 100 % methanol as mobile phase. Chromatographic runs were performed isocratically at a flow rate of 1.0 mL/min and the column temperature was kept constant at 28 °C.

The eluted compounds were detected spectrophotometrically at 245 nm (Vit C), 292 nm (Vit E) and 325 nm (retinyl acetate) and fluorometrically at 325 nm/475 nm excitation/emission wavelengths (Vit A and retinyl acetate). Chromatograms were analysed by the Galaxie Chromatography Workstation software (version 1.9, Varian Inc.). Sample concentrations were calculated from peak areas by a linear calibration model. The limits of detection, calculated as the analyte concentration corresponding to a signal-to-noise (S/N) ratio of 3, were 0.6 μmol/L, 0.1 μmol/L and 2 μmol/L for Vits C, A and E, respectively. A correction factor was applied to Vit C results to correct for dilution by DTE. The chemicals used were of the highest purity available (Sigma-Aldrich) and the water was ultrapure (Milli-Q water filtration system, Millipore Spa, Rome, Italy).

### RBP and total lipids assays

Plasma RBP was determined by a commercial nephelometric assay (BNII, Siemens Healthcare Diagnostics, Tarrytown, NY, USA). Cholesterol and triglycerides were determined by local clinical chemistry laboratories by means of commercial enzymatic methods. Total lipids were calculated as the sum of cholesterol and triglycerides.

### Data analysis

The statistical power was verified by the Authors and Biostatistic Specialist of Ethic Committee. All blood values at the end of the dialysis session were corrected for hemoconcentration. Results are expressed as means and standard deviations - or medians and interquartile range depending on the distribution’s normality - for continuous data and as frequency for categorical data. The assumption of normality was checked by the Shapiro-Wilk test. Vitamin levels below the method LOD were assigned a value of ½ LOD. For all the variables but AOPP and TAS, the long-term effects of the treatments were compared by either CROS-analysis [39] (HFR-S vs HFR) or repeated measures ANOVA (HFR vs HFR-S vs run-in) using either parametric or non-parametric tests as appropriate. For AOPP and TAS, paired and unpaired *t*-test (or, when appropriate, their non-parametric equivalents) were used for comparison within arms and between arms, respectively. As for the acute effects, the percent reduction ratios (RR% = [start session value – end session value] *100/start session value) of the different treatments were compared by either ANOVA or ANCOVA, the latter being used in case of significant correlation between pre-dialysis levels and intra-dialytic losses. Only values ≥ LOD were entered into RR% calculation. Associations were assessed by Spearman rank correlation test. Extreme outliers (more than 3 inter-quartile ranges) were excluded from the statistical tests. *P* < 0.05 were considered significant and all tests were 2-tailed. Statistical analysis and data plotting were performed by using SigmaPlot ® 12.0 (Systat Software Inc.).

## Results and discussion

Patient demographics and clinical characteristics at baseline are reported in Table 2. Study arms were well balanced and no significant biases were introduced by study dropouts at any time (data not shown). The operating conditions and adequacy parameters of the different extracorporeal treatments adopted in the study are reported in Table 3.

**Table 2:** Personal and clinical data of patients at enrollment

	All	Arm A	Arm B
*n*	41	21	20
Age (years)	70 (61–76)	73 (56–79)	67 (65–73)
Dialysis vintage (months)	69 (35–153)	107 (53–164)	60 (29–135)
Gender	M = 26 (63 %)	M = 12 (57 %)	M = 14 (70 %)
	F = 15 (37 %)	F = 9 (43 %)	F = 6 (30 %)
Albumin (mg/dL)	3.6 (3.4–3.9)	3.6 (3.4–4.0)	3.7 (3.5–3.9)
Mean Body weight (kg)	M = 63.5 ± 9.6	M = 63.2 ± 7.9	M = 63.8 ± 11.2
	F = 56.5 ± 10.5	F = 53.6 ± 10.2	F = 60.7 ± 10.2
BMI	M = 23.7 ± 3.4	M = 24.1 ± 2.3	M = 23.3 ± 4.2
	F = 23.8 ± 4.4	F = 23.0 ± 4.1	F = 25.0 ± 4.8
Cardiovascular disease	59 %	62 %	55 %
Hypertension	78 %	81 %	75 %
Diabetes	22 %	19 %	25 %
Charlson Comorbidity Score	6 (6–7)	6 (5–7)	6 (6–7)
Previous dialysis treatment	BHD = 15 (37 %)	BHD = 8 (38 %)	BHD = 7 (35 %)
	HDF = 10 (24 %)	HDF = 4 (19 %)	HDF = 6 (30 %)
	HFR = 16 (39 %)	HFR = 9 (43 %)	HFR = 7 (35 %)

Continuous data are expressed either as mean ± SD or, in case of not normally distributed data, as median (IQR)

**Table 3:** Operating conditions and adequacy parameters of the different dialysis treatments adopted in the study

	ol-HDF (run-in)	HFR-S	HFR
Session length (min)	233 ± 18	235 ± 25	233 ± 16
Qb (mL/min)	313 ± 31	312 ± 31	301 ± 32
Qd (mL/min)	500	500	500
Body weight loss (kg/session)	2.8 ± 0.8	2.7 ± 1.1	2.8 ± 0.9
(Re)infusion volume (L/session)	14.9 ± 2.5	12.9 ± 2.9	13.4 ± 4.1
(Re)infusion rate (mL/min)	64.2 ± 9.6	54.9 ± 13.4	57.5 ± 13.2
*eq*Kt/V	1.22 ± 0.25	1.17 ± 0.32	1.12 ± 0.23
ePCR (g/Kg/day)	1.4 ± 0.6	1.07 ± 0.54	1.08 ± 0.35

Data are expressed as mean ± SD (*n* = 41, 50 and 50 for ol-HDF, HFR-S and HFR, respectively)

Patients’ biochemical data at the end of the run-in and of each treatment period are reported in Table 4. At the end of the run-in, most of the patients had low Vit. C, high Vit. A and normal Vit. E levels; RBP values were also higher than in non-uremic subjects resulting in lower-than-normal Vit. A/RBP molar ratios; AOPP values were in the normal range, while TAS values were higher-than-normal. For all parameters but AOPP, the levels we found were in agreement with those previously reported for uremic subjects [3, 8, 11, 40–42]. At difference, AOPP levels were significantly lower than it was previously reported for these patients [36]. Although this discrepancy could be due, at least in part, to the lack of standardized measurements for AOPP [43], this result suggests that our cohort was characterized by a low level of oxidative damage at baseline. Given that a significant association has been reported between malnutrition and oxidative stress in ESRD [44], the above evidence is in good agreement with and could be partially explained by the good nutritional status of enrolled patients (Table 2). Moreover, the use in all the participating centers of ultrapure dialysate locally produced by biosmosis could also have played a role in decreasing dialysis-related oxidative stress in our patients’ cohort [45].

**Table 4:** Pre-dialysis levels of antioxidant vitamins and oxidative stress biomarkers at the end of run-in and of either HFR-S or HFR treatments

	End of run-in	End of HFR-S	End of HFR
Vitamin C (μmol/L)	20.0 (11–32)	21.5 (12–43)	20.0 (10–31)
Vitamin A (μmol/L)	2.8 ± 1.4	2.6 ± 1.0^a^	3.0 ± 1.2
Vitamin E (μmol/L)	20.2 ± 5.2	20.9 ± 5.9	20.9 ± 5.7
RBP (μmol/L)	5.8 ± 1.4	6.5 ± 1.6^a, b^	6.9 ± 1.6^b^
Total lipids (mmol/L)	5.6 ± 1.2	6.1 ± 1.7	6.3 ± 1.7
Vitamin A/RBP (μmol/μmol)	0.47 ± 0.17	0.40 ± 0.11^b^	0.42 ± 0.13^c^
Vitamin E/lipids (μmol/mmol)	3.6 ± 0.7	3.6 ± 0. 9	3.4 ± 0.8
AOPP (μmol/L)	28.4 (25–37)	21.7 (20–26)^d^	-
	35.8 (24–45)	-	27.3 (21–34)^d^
TAS (mmol/L)	1.88 (1.7–2.2)	1.41 (1.3–1.6)^d^	-
	1.99 (1.8–2.2)	-	1.43 (1.4–1.5)^d^

For all parameters but AOPP and TAS, only patients who completed the study were included in the analysis and cumulative data of the two treatment periods in HFR-S/HFR are represented. For AOPP and TAS only data of the first treatment period were available, hence all patients who reached this stage of the study were included in the analysis. Data are expressed either as mean ± SD or, in case of not normally distributed data, as median (IQR)

^a^ = *p* < 0.05 vs HFR, *n* = 29, Cross-analysis [39]

^b^ = *p* < 0.01 and ^c^ = *p* < 0.05 vs End of run-in, *n* = 29, ANOVA for repeated measures

^d^ = *p* < 0.01 vs End of run-in, *n* = 14 (HFR-S) or 16 (HFR), Wilcoxon Signed Rank Test

**Fig. 3:**
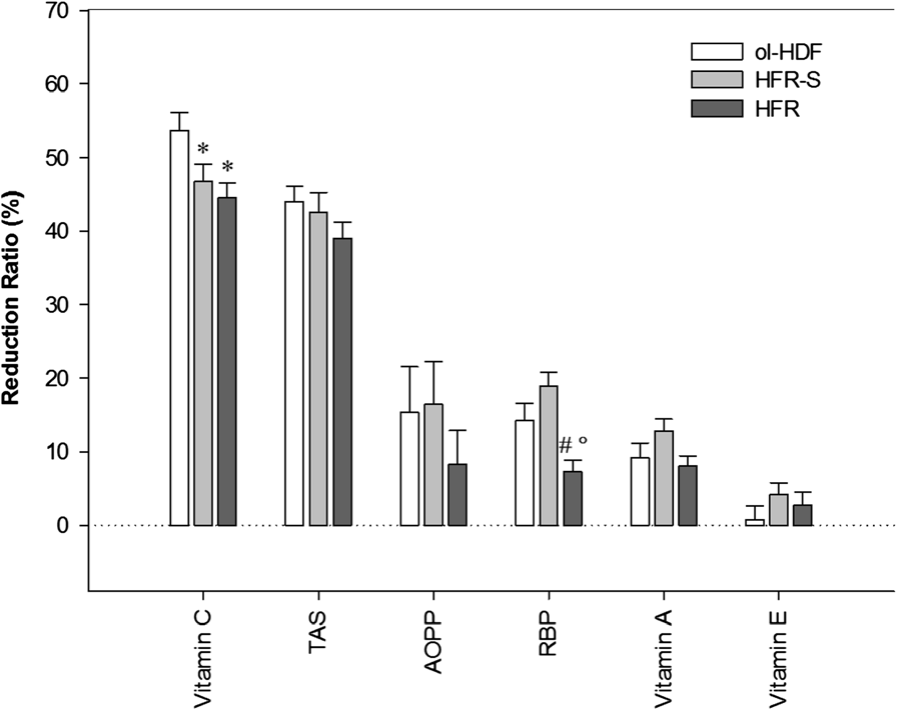
Reduction ratios of the studied variables at the end of ol-HDF (*n* = 41), HFR-S (*n* = 50) and HFR (*n* = 50) dialysis sessions. For AOPP and TAS the numbers of studied sessions are 30, 28 and 32 for ol-HDF, HFR-S and HFR, respectively. Each dialysis session at each time of the study was included in the analysis. Data are expressed as means ± SE (error bar). * = *p* < 0.01 vs ol-HDF; # = *p* < 0.05 vs ol-HDF; ° = *p* < 0.01 vs HFR-S; ANOVA (RBP, Vitamin A, Vitamin E) or ANCOVA (Vitamin C, TAS, AOPP)

At the end of the study, no significant differences were found between HFR-S and HFR as for their long-term effects on Vit C, Vit E, Vit E/lipids, AOPP and TAS levels (Table 4). At difference, significantly lower levels of Vit A and of its plasma carrier, RBP, were measured after HFR-S than after HFR, the Vit A/RBP ratio being not significantly different between the two treatments (Table 4, superscript asterisks). Accordingly, in the short-term, the only significant difference between HFR-S and HFR was the RBP RR% which was higher in HFR-S (Fig. 3). The present results suggest that HFR-S and HFR are equivalent in terms of effects on patients’ oxidative status. The modest difference in Vit A plasma levels did not significantly affect the plasma antioxidant capacity and is easily explained by the difference in the convective permeabilities of the two techniques: the higher convective permeability of HFR-S facilitates the removal of both RBP (MW = 21.2 kDa) and RBP/Vit A complexes from plasma leading to the decrease of the circulating levels of both the protein and its ligand. In fact, at difference of healthy subjects in whom virtually all Vit. A/RBP complexes are bound to prealbumin so preventing the renal loss of this vitamin, Vit. A/RBP complexes unbound to prealbumin are significantly increased in ESRD [40], allowing Vit. A passage trough high-flux membranes and its loss in the outflow dialysate. The evidence that the Vit A /RBP ratio was unchanged between the two treatments substantiates the above interpretation.

It has been demonstrated that the removal of middle-large uremic solutes by convective techniques contribute to decrease oxidative stress in haemodialysis patients [16]. Thus, we can conclude that the beneficial effects on oxidative stress produced by using high-flux membranes in HFR are not significantly improved by using hyper high-flux membranes in HFR-S, ie by further increasing the convective permeability. It could be argued that the low starting levels of AOPP might have decreased the power of the present study and that a higher number of subjects as well as a longer observation period would allow significant differences between HFR and HFR-S to emerge. Although we cannot rule out this possibility, the evidence that AOPP levels significantly decreased during the course of both HFR-S and HFR without significant differences between the two treatments (Table 4, superscript letters) makes this hypothesis unlikely. In fact, as compared to the values reached at the end of the run-in, both treatments induced a significant decrease of both AOPP and TAS and an increase of RBP (but not of vit. A) levels (Table 4, superscript letters). These long-term changes cannot be merely explained by differences in AOPP, TAS and RBP RR%s: actually, the RR%s measured during the run-in sessions (ol-HDF) were similar to those measured during HFR-S and HFR sessions, with the sole exception of RBP, whose RR% was significantly lower in HFR than in either HFR-S or ol-HDF, without significant differences between ol-HDF and HFR-S (Fig. 3). Thus, since RBP levels increased over time during both HFR and HFR-S treatments, it is unlikely that the differences in RR%s play a significant role in this phenomenon. Instead, the above results suggest that, as compared to ol-HDF, during both the re-infusive treatments an homeostatic response took place, consisting of 1) a progressive reduction of systemic oxidative stress accompanied by an adaptative reduction of the plasma antioxidant potential, and 2) an increase of RBP circulating levels.

**Fig. 4:**
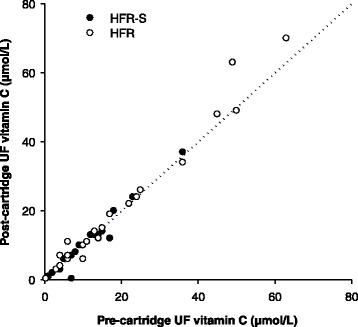
Comparison between the vitamin C levels of pre- and post-cartridge UFs during HFR-S and HFR dialysis sessions. Dotted line = identity line

In our study, ol-HDF, HFR-S and HFR used the same machines, same ultrapure dialysate and same PES membranes, although that used in HFR-S had a higher convective permeability (see Table 1). Thus, in our study the main difference between ol-HDF and HFR-S/HFR consisted in the UF re-infusion. The re-infusion solution is the same plasmatic water of the patient purified on the sorbent cartridge; the composition of this solution depends on the sorbent adsorption capability which is virtually zero with respect to albumin and amino acids and significantly different from zero with respect to some uremic toxins and inflammatory citokines [18–24]. In the present study we show for the first time that the sorbent cartridge does not significantly adsorb Vit. C, since no significant differences were found between the Vit.C concentration of pre- and post cartridge UF neither in HFR-S nor in HFR (ns Wilcoxon Signed Rank Test, *n* = 22 and 26, respectively, Fig. 4). That means that the ultrafiltered Vit C can be entirely returned to the circuit, thereby its intra-dialytic loss is reduced. It has been reported that during a HDF session, both convective and diffusive losses of Vit. C occur, both potentially contributing to the oxidative stress in haemodialysis patients [6]. Accordingly, in the present study we show that the two re-infusive techniques allowed to reduce Vit C RR% by about 15 % as compared to ol-HDF (Fig. 3). Despite the sparing effect produced by each HFR-S/HFR session, we did not find any significant long term increase of Vit. C levels during the treatments (Table 4). This may be due, at least in part, to the high intra- and inter-subject variability of the plasma levels of this vitamin. Nonetheless, it cannot be ruled out that the sparing of Vit. C – and possibly of other small hydrophilic plasma components endowed with antioxidant/nutritional properties – may have played a role in the long-term improvement of oxidative status produced by the two re-infusive treatments.

As for RBP, the present results suggest that, as compared to ol-HDF, both HFR-S and HFR activate endogenous mechanisms of RBP plasma level modulation, likely due to the stimulation of its *de novo* synthesis. The RBP increase was not associated with a corresponding Vit. A increase, resulting in a net reduction of the Vit. A/RBP ratio (Table 4). This result was rather unexpected, since RBP is physiologically secreted bound to retinol. However, in uremic patients the retinol content of the liver is dramatically reduced [46], thus giving a possible explanation for the above result.

At this time we can only speculate about the biological mechanisms underlying the observed increase of RBP plasma levels. From a physiological point of view, RBP behaves as a negative acute phase protein, ie both inflammation and protein malnutrition depress its hepatic synthesis [47–50]. It is known that hemodialysis patients are at higher risk of both inflammation and malnutrition, a large part of them exhibiting an activated acute phase response [51]. Hence, RBP increase might be a consequence of an improvement of patient inflammatory/nutritional status. A previous study did not find significant differences between the circulating levels of some inflammatory markers in HFR and ol-HDF, thus making this hypothesis unlikely [29]. However, it is worth underlying that in the present study RBP increase was accompanied by a significant improvement of patients’ oxidative status and that oxidative stress and inflammation share common pathogenic mechanisms [52]. From a pathological point of view, it should be taken into consideration that in ESRD free RBP and Vit A/RBP complexes are chronically increased, mainly due to the impairment of RBP renal catabolism [11, 40]. Based on an experimental model of AKI, it has been also proposed that a peripheral regulatory signal, normally removed by the kidney, might accumulate in the circulation and upregulate the hepatic release of Vit A/RBP complexes [53]. Thus one can hypothesize that this supposed regulatory signal accumulates in plasma of CKD patients, from where it might be removed by convective transport and, only in case of HFR(−S), be returned to the patient by means of UF re-infusion. In such a way, the stimulus to RBP hepatic release would be lower in ol-HDF than in either HFR-S or HFR. Anyway, regardless of the mechanisms behind the RBP level increase, higher RBP and Vit. A values in hemodialysis patients are associated with a better prognosis, lower values - even still higher than normal values - being strongly associated with higher mortality and cardiovascular risk [12, 13]. With this in mind and considering also that convective dialysis compared to purely diffusive dialysis is supposed to reduce cardiovascular mortality [54], further studies should be undertaken to i) investigate on the long-term impact of different convective techniques on patients’ survival and cardiovascular risk, and ii) better define, if any, the distinctive roles of Vit. A and RBP in the phenomenon.

Finally, we found that, irrespective of the dialysis treatment adopted, i) Vit. C levels were negatively related to the patients’ age and positively related to the vit. A levels (*p* < 0.01), and, as expected, ii) Vit._s_ A and E levels were highly correlated to their respective plasma carriers, RBP and total lipids (*p* < 0.001,Table 5). A strong negative association between Vit. C and age in older people has been previously reported for the general population [55]. Hence, it is likely that physiological mechanisms linked to the elderly rather than to the kidney disease *per se* are responsible for the above association. The strong association between Vit. C and Vit. A is instead rather surprising since these vitamins are supposed to exert their antioxidant activity in different biological environments; in fact, while Vit. C is a water-soluble antioxidant, Vit. A is a lipid-soluble antioxidant, being significantly less effective than Vit. E in scavenging aqueous radicals [56]. In the general population only a weak interaction was found between Vit. C and Vit. A while a stronger association was found between Vit. C and Vit. E, likely explained by their close physiological interaction [57]. At difference, in our patients Vit. C was significantly related only to Vit. A. If mechanisms other than radical scavenging mediate Vit. C/Vit. A association in ESRD patients deserves further investigation and is beyond the aims of the present study.

**Table 5:** Significant results of bivariate correlation analysis of antioxidant vitamins and oxidative stress biomarkers pre-dialysis levels at the end of run-in and after a six-months treatment period in HFR-S or HFR

	End of run-in	End of HFR-S	End of HFR
Vitamin C vs age	−0.626*** (31)	−0.543** (30)	−0.553** (30)
Vitamin C vs Vitamin A	0.476** (31)	0.529** (30)	0.510** (30)
Vitamin A vs RBP	0.841*** (32)	0.613*** (31)	0.616*** (30)
Vitamin E vs total lipids	0.689*** (31)	0.642*** (27)	0.639*** (27)

Analysed parameters included: age, dialysis vintage, Vit C, Vit A, Vit E, RBP, total lipids, Vit A/RBP, Vit E/total lipids, AOPP, TAS. All patients that completed at least the first treatment period were included in the analysis

Data are expressed as rs (n), Spearman correlation test

*p < 0.05; **p < 0.01, ***p < 0.001

Limitations of the present study were the relatively small sample size, the ethnicity of enrolled patients (all from Sardinia, an Italian island housing genetically homogenous populations with traditional living habits) and the lack of measurement of inflammation and uremic retention biomarkers. Further studies overcoming the above limitations will allow to strengthen and expand on the present results.

## Conclusion

Kidney failure is associated with oxidative stress. Hemodialysis may contribute to increase oxidative stress of ESRD patients due to the activation of inflammatory pathways and to the loss of small water-soluble antioxidants. Convective dialysis modalities can reduce the burden of oxidative stress of hemodialysis patients by allowing the removal of large pro-oxidant uremic solutes from plasma. Unlike ol-HDF, which is a mixed convective-diffusive method, HFR-S and HFR exploit in sequence convection, adsorption and diffusion. Thanks to the adsorption step, UF is purified by toxic molecules and it can be re-infused to the patient. Since other UF components such as albumin, amino acids and Vit C are not adsorbed, HFR architecture allows to increase convective permeability without the risk of losing useful substances.

In the present study, we show that the higher convective permeability of HFR-S with respect to HFR did not produce additional beneficial effects on the oxidative status of ESRD patients, the only significant difference being a long-term decrease of Vit. A due to the more efficient removal of its plasma carrier, RBP. Further studies are needed to investigate whether the more efficient removal of large molecules by HFR-S produces additional beneficial effects over HFR in term of (micro)inflammation and uremic toxins retention.

In comparison to ol-HDF, both the re-infusive techniques allow to significantly reduce the intradialytic loss of Vit. C and, in the long-term, improve the patients’ oxidative status and increase RBP plasma values, all effects which might have a potentially positive impact on patients’ cardiovascular morbidity and mortality.

Nutrient supplementation is often used to provide malnourished haemodialysis patients with essential aminoacids, vitamins and minerals and/or to counteract the loss of useful substances upon high-convection dialysis techniques. HFR-S and HFR, by returning his own plasmatic water back to the patient, can be a way to restore a more physiological status without additional therapies. Moreover, both the financial and technical costs of nutrient supplementation make HFR definitely more advantageous over other convective techniques with similar depurative capacity. Finally, HFR architecture paves the way for potential, further increase of convective permeability, an improvement otherwise entailing severe loss of plasma proteins which must then be restored by additional infusional therapies.

Ol-HDF has been reported to produce clinical and survival benefits with respect to HD. Nonetheless ESRD patients on convective dialysis still show high levels of inflammation, oxidative stress and high cardiovascular morbidity and mortality. HFR-S and HFR could represent a new biotechnological response potentially able to reduce side effects and complications. The long-term impact of these new techniques on patients’ survival and cardiovascular risk need to be evaluated.

## Abbreviations

AOPP, advanced oxidation protein products; DTE, dithioerythritol; ESRD, end stage renal disease; HFR, hemodiafiltration with on-line endogenous reinfusion; HFR-S, HFR-Supra; ol-HDF, on-line hemodiafiltration; PES, polyethersulfone; RBP, retinol binding protein; RR, reduction ratio; SDVB, styrene divinylbenzene; TAS, total antioxidant status; UF, ultrafiltrate; Vit A, vitamin A; Vit C, vitamin C; Vit E, vitamin E
